# Supplementary material for: Measuring threshold and latency of motion perception on a swinging bed
Source: PLoS One. 2021 Jul 9;16(7):e0252914. doi: 10.1371/journal.pone.0252914 (PMC8270192; doi:10.1371/journal.pone.0252914)
Supplement: Attachment — Submitted filename: response to reviewers 2.docx [file pone.0252914.s004.docx]

Response to reviewers:

You have satisfactorily addressed all the issues raised by the reviewers.
However, I have one comment and one remark, both minor.

Q1- In the summary, you should specify that the peak is a position one.

A1- This information has now been added to the abstract.

Q2- In the discussion you should add a comment on the fact that the subject can (or cannot) use information from the beep in the synchronicity task.
Indeed, auditory system is able to detect when the sound source is moving away or approaching using Doppler effect or changes in sound intensity. As velocity at the peak position is zero, the subject knows that the beep is synchronous to the peak position only if he does not perceive the sound source moving. I guess 5ms is too short for a beep to give movement information, but you should comment this (with references).

A2- We totally agree. This could have been a source of information but changes in the sound spectrum or the doppler effect are minimal for a 5 ms beep (0.16% of the oscillation period). We have now added this discussion together with 3 references as follows (lines 339-343): “Another issue is the detection of the sound source movement relative to the head position by monuaral (spectral changes, doppler effect) [33] or binural functions (interaural time and intensity differences) [34]. These effects are sensitive to signal duration [35]. Considering the shortness of the sound stimuli (5 ms, 0.16% of the oscillation period), this effect can be considered as negligeable.”

References:

33- Grothe B, Pecka M, McAlpine D. Mechanisms of sound localization in mammals. Physiol Rev. 2010;90:983-1012. doi: 10.1152/physrev.00026.2009.

34- Baumann C, Rogers C, Massen F. Dynamic binaural sound localization based on variations of interaural time delays and system rotations. J Acoust Soc Am. 2015 Aug;138(2):635-50. doi: 10.1121/1.4923448.

35- St George BV, Cone B. Perceptual and Electrophysiological Correlates of Fixed Versus Moving Sound Source Lateralization. J Speech Lang Hear Res. 2020 Sep 15;63(9):3176-3194. doi: 10.1044/2020_JSLHR-19-00289.
